# Mechanistic Links Underlying the Comorbidity of Osteoporosis and Osteoarthritis: Cell Fate Plasticity Driven by the Subchondral Bone Microenvironment

**DOI:** 10.3390/ijms27135757

**Published:** 2026-06-25

**Authors:** Jian Zhang, Bingbing Chen, Qianqian Yang, Heguo Yan, Niqin Xiao, Yundong Xu, Sanjin Zeng, Shengyi Zhao, Rong Wang, He Qian, Zhaohu Xie, Jing Xie, Zhaofu Li

**Affiliations:** 1 The First Clinical Medical College, Yunnan University of Chinese Medicine, No. 1076 Yuhua Street, Chenggong District, Kunming 650500, China; 17860503891@163.com (J.Z.); qqy0802@126.com (Q.Y.); yanheguo1990@163.com (H.Y.); xnq2022@126.com (N.X.); xuxu1204@126.com (Y.X.); 2 College of Basic Medical Sciences, Yunnan University of Chinese Medicine, No. 1076 Yuhua Street, Chenggong District, Kunming 650500, China; chenbing5682025@126.com (B.C.); 15580760163@163.com (S.Z.); zsy89858763@outlook.com (S.Z.); 15750131998@163.com (R.W.); qianheyucm@163.com (H.Q.); xiezhaohu@ynucm.edu.cn (Z.X.)

**Keywords:** osteoporosis, osteoarthritis, comorbidity, osteochondral unit, subchondral bone, microenvironment, cell fate plasticity

## Abstract

Osteoporosis (OP) and osteoarthritis (OA) are two common degenerative musculoskeletal disorders associated with aging and are traditionally classified and managed as distinct disease entities. Emerging evidence suggests that OP and OA may share bidirectional associations and common biological mechanisms, and that under specific pathological conditions they may develop into a mutually reinforcing comorbid state. The comorbidity of osteoporosis and osteoarthritis (OP–OA) is not a simple superimposition of bone loss and cartilage degeneration; rather, it represents a disorder of the osteochondral unit centered on disruption of the subchondral bone microenvironment. Alterations in the structural strength, remodeling dynamics, vascular and neural status, and bone marrow lesions of subchondral bone collectively reshape the local microenvironment, thereby directly affecting mechanical signal transmission and cellular behavior within the joint. Focusing on the subchondral bone microenvironment as the central pathological nexus, this review systematically summarizes how mechanical imbalance, aberrant bone remodeling, inflammatory activation, metabolic dysregulation, and cellular senescence jointly remodel the local niche in OP–OA comorbidity. These microenvironmental changes further induce phenotypic remodeling and fate deviation of bone marrow mesenchymal stem cells, bone remodeling-related cells, osteoimmune cells, and chondrocytes. On this basis, we integrate the regulatory roles of developmental signaling, mechanotransduction pathways, and inflammatory–immune signaling networks, and propose that microenvironment-driven cell fate plasticity may serve as a key mechanistic hub promoting the initiation and progression of OP–OA comorbidity as well as the persistent destabilization of the osteochondral unit. This perspective may help overcome the limitations of current studies that address OP and OA separately, and may provide a theoretical framework for early identification and stratification, biomarker discovery, and combined precision-targeted interventions for this comorbid condition.

## 1. Introduction

Osteoporosis (OP) and osteoarthritis (OA) are among the most prevalent degenerative skeletal disorders associated with aging. Both conditions are characterized by high prevalence and substantial disability, and their global disease burden has continued to increase in recent years [1,2]. Traditionally, OP and OA have been investigated along separate research trajectories and have even been conceptualized as disorders with contrasting mechanical and metabolic features. OP is primarily characterized by reduced bone mass, deterioration of bone microarchitecture, and an increased risk of fragility fractures, whereas OA is commonly regarded as a chronic degenerative joint disease driven by articular cartilage degeneration and abnormal mechanical loading. However, accumulating evidence indicates that OP and OA are not entirely distinct entities. Rather, they share several risk factors, including inflammation, advanced age, and metabolic dysregulation, as well as overlapping genetic architectures, biological mechanisms, and potential causal relationships [3,4]. These findings suggest that OP and OA may have a complex comorbid basis and, in certain populations, may manifest as osteoporosis–osteoarthritis (OP–OA) comorbidity [5].

In OP–OA comorbidity, degeneration of articular cartilage appears to be more pronounced, as reflected by more extensive cartilage fibrosis, deep fissuring, and focal full-thickness cartilage loss. Meanwhile, the subchondral bone undergoes aberrant bone remodeling, trabecular disorganization, and markedly increased bone turnover, ultimately leading to aggravated subchondral bone sclerosis [6]. Subchondral bone not only contributes to load transmission and mechanical buffering but also shapes the local microenvironment through bone remodeling, vascular and neural remodeling, inflammation, and metabolic regulation. Through these mechanisms, subchondral bone influences cartilage homeostasis and pain generation and may participate in driving disease progression from the early stages of OA [7,8]. Evidence from human specimens further suggests that, in the setting of OA combined with OP, cartilage degeneration and aberrant subchondral bone remodeling coexist, supporting a central role of the osteochondral interface in this comorbid condition [9]. Based on this rationale, the present review adopts “cell fate plasticity driven by the subchondral bone microenvironment” as its central perspective. We systematically review the clinical and pathological basis of OP–OA comorbidity, abnormal subchondral bone remodeling, and the associated alterations in multiple cell fates, while integrating the key signaling networks involved. This framework may provide new insights into trans-tissue interactions within the osteochondral unit and inform precision therapeutic strategies for OP–OA comorbidity.

## 2. Clinical and Pathological Basis of OP–OA Comorbidity

### 2.1. Epidemiological Co-Occurrence and Overlapping Risk Factors

Recent genetic epidemiological evidence and clinical observations suggest that OP and OA are linked by complex comorbid relationships in the contexts of aging, postmenopausal status, and metabolic abnormalities [10,11]. Studies have shown that the prevalence of OP among patients with OA is not negligible and may vary substantially according to study design, anatomical site of measurement, and population characteristics. Therefore, a simplistic distinction based solely on “bone sclerosis” or “low bone mass” is insufficient to explain the comorbid phenomenon [12]. From the perspective of risk factors, postmenopausal women experience bone loss due to aging and estrogen deficiency. Cortical bone deterioration may lead to uneven mechanical loading of intra-articular cartilage, disruption of trabecular microarchitecture, and even collapse of the articular surface. These changes disturb the biomechanical equilibrium within the joint cavity and impair the transmission of load-induced stress from cartilage to subchondral bone. Evidence from animal models further suggests that reduced muscle strength and abnormal lower-limb alignment may decrease joint stability and increase imbalanced interarticular friction, thereby accelerating cartilage wear and contributing to the onset of OA [13]. Conversely, during the middle stages of OA progression, osteophyte formation and impaired cartilage function may lead to dysregulated bone metabolism in the subchondral region, which can subsequently contribute to secondary OP [14]. Thus, OP–OA comorbidity essentially represents a vicious pathological cycle. Multiple shared risk factors may simultaneously act on bone remodeling imbalance and disruption of joint homeostasis, allowing OP and OA to arise within the same individual on a shared biological background. Accordingly, OP–OA comorbidity should be understood as a composite musculoskeletal phenotype driven by common risk factors.

### 2.2. Imaging Features and Histological Evidence

Imaging and histological studies indicate that the osteochondral interface is a key lesion site in OP–OA comorbidity, particularly the subchondral bone plate, trabecular bone, and bone marrow cavity microenvironment. Quantitative magnetic resonance imaging (MRI) analyses have shown that, in patients with OA combined with OP, clinical symptoms are more strongly associated with bone marrow lesions and subchondral bone alterations than with cartilage thickness. This finding suggests that symptoms in these patients may originate predominantly from subchondral bone pathology rather than from cartilage wear alone [15]. Studies of peri-knee bone mineral density have also shown that postmenopausal patients with OA and OP exhibit periarticular bone mineral density distributions that differ from those observed in patients with OA alone. These distributions are associated with Western Ontario and McMaster Universities Osteoarthritis Index (WOMAC) scores, radiographic severity, and lower-limb alignment parameters, indicating that local bone mass and joint pathology are not independent processes. At the histological level, pathological grading of subchondral bone changes may progress in parallel with the severity of cartilage degeneration. These changes include thickening of the subchondral bone plate, disorganized trabecular orientation, and abnormalities of the calcified cartilage layer, suggesting that cartilage degeneration and bone remodeling occur as coupled processes [16]. In early rabbit models of OP–OA comorbidity, improvement of subchondral bone microarchitecture and remodeling parameters using the bone anabolic agent parathyroid hormone (PTH) has been shown to improve subchondral bone density and bone damage, thereby preventing cartilage injury [17]. Micro-computed tomography (micro-CT) assessment of femoral head specimens from patients with OA further revealed that, when OP and OA coexist, the microstructural and mineralization characteristics of subchondral bone exhibit marked regional and compartment-specific differences. In both OP and OA, the degree of mineralization in subchondral bone is higher than that in the subchondral plate, and these compartmental differences vary according to the severity of cartilage degeneration [18]. These findings further demonstrate that the comorbid state is not simply characterized by either “osteoporosis” or “bone sclerosis”; rather, it represents a complex structural alteration involving low bone mass, focal remodeling, and abnormal mineralization.

### 2.3. Phenotypic Heterogeneity and Clinical Relevance

OP–OA comorbidity is not a single homogeneous disease state but instead resembles a clinical phenotypic spectrum with marked spatiotemporal heterogeneity. This heterogeneity may be reflected in bone mineral density, patterns of subchondral bone remodeling, pain severity, and functional outcomes. Analyses from the Osteoarthritis Initiative database suggest that patients with OA can be stratified into distinct subgroups according to clinical and structural characteristics. Patients with concomitant OP appear to exhibit a bone-related phenotype dominated by subchondral bone abnormalities, in which pain and functional impairment are more strongly associated with osseous changes than with cartilage damage alone [19]. Studies focusing on OA clinical phenotypes, molecular endotypes, and treatment stratification have further emphasized that differences in bone remodeling, inflammation, metabolism, and pain mechanisms are important contributors to the heterogeneity of OP–OA comorbidity [20]. This heterogeneity has clear clinical significance. Increased subchondral bone fragility may progress to subchondral insufficiency fracture, leading to articular surface collapse, aggravated pain, and secondary deterioration of joint structure [21]. Therefore, OP–OA comorbidity not only shapes structural phenotypes but may also influence clinical outcomes and management strategies. Its management should move beyond single-disease paradigms toward stratified assessment and precision intervention based on the osteochondral unit.

## 3. Aberrant Subchondral Bone Remodeling in OP–OA Comorbidity

### 3.1. Structural and Mechanical Imbalance at the Osteochondral Interface

The osteochondral interface consists of hyaline cartilage, the tidemark, calcified cartilage, and the subchondral bone plate, and serves as a key functional unit for mechanical load transmission and molecular exchange within the joint [22,23]. Under physiological conditions, hyaline cartilage displays a well-organized zonal architecture. The superficial zone is enriched in type II collagen (COL2) and lubrication-associated molecules, enabling resistance to shear stress, whereas the deep zone absorbs compressive loads through vertically aligned collagen fibers [24]. The tidemark, which demarcates the boundary between deep cartilage and calcified cartilage, is continuous, distinct, and regular in healthy joints and represents an important histological marker of cartilage maturation and interface stability [22]. Calcified cartilage forms a functional gradient layer between cartilage and bone, and its continuous stiffness transition helps dissipate stress and reduce stress concentration [25].

During OP–OA comorbidity, this gradient architecture is initially disrupted by subchondral bone fragility. Unlike the focal sclerosis commonly observed in late-stage isolated OA, the comorbid state is often characterized by reduced bone mass, impaired material properties of the subchondral bone plate, and insufficient trabecular support beneath the osteochondral interface. These changes prematurely weaken the mechanical foundation of the osteochondral interface [26]. Studies have shown that the mechanical and material properties of the OP-associated subchondral bone plate are significantly inferior to those observed in OA, and the resulting decline in structural support can directly alter the loading environment of the overlying cartilage [27]. On this weakened structural basis, OA-related abnormal loading is more likely to concentrate in the deep cartilage and calcified cartilage layers, shifting stress distribution at the interface from a physiological gradient pattern to pathological focal concentration [28]. As the disease progresses, the calcified cartilage layer may undergo thickening, pathological mineralization, and formation of multiple tidemarks, indicating disruption of the original continuous transition structure [29]. Although pathological calcification can increase local stiffness, it compromises the buffering capacity of the interface, thereby promoting stress concentration, crack propagation, and microdamage accumulation [25]. Concurrently, the subchondral bone plate undergoes marked remodeling, characterized by plate thickening, increased hardness, disorganized trabecular microarchitecture, and abnormally active bone turnover [30]. Therefore, osteochondral interface imbalance in OP–OA comorbidity can be conceptualized as a sequential and mutually reinforcing process: OP first compromises the osseous support and buffering capacity beneath the interface, whereas OA subsequently promotes abnormal mineralization, stress concentration, and interface remodeling. Together, these processes establish a vicious cycle in which structural degeneration and mechanical imbalance amplify one another.

### 3.2. Spatiotemporal Heterogeneity of Subchondral Bone Remodeling Dynamics

In the context of OP–OA comorbidity, subchondral bone remodeling is not a uniform or static process but instead exhibits marked spatiotemporal heterogeneity. A central feature of this process is that OP-induced low bone mass and bone fragility alter the onset, rhythm, and spatial distribution of OA-associated remodeling. Experimental studies have shown that when OA develops secondary to OP, subchondral bone exhibits earlier high-turnover remodeling and microstructural damage, which can directly aggravate subsequent cartilage degeneration [31]. Other studies have demonstrated that patients with OP–OA comorbidity show more severe deterioration of subchondral trabecular microarchitecture than patients with OA alone, characterized by reduced plate-like trabeculae, increased rod-like structures, and an association with more severe cartilage damage. These findings suggest that remodeling in the comorbid state is not simply homogeneous sclerosis, but rather aberrant remodeling based on destabilized osseous support [32].

From a temporal perspective, subchondral bone remodeling shows typical stage-dependent characteristics. In isolated early OA, enhanced bone resorption, reduced trabecular bone mass, and increased porosity of the subchondral bone plate are commonly observed, followed by gradual progression toward plate thickening and focal sclerosis [33,34]. However, in the presence of OP, this process is often initiated earlier and becomes more unstable. Early-stage changes are dominated by osteoclast-mediated high bone turnover and trabecular deterioration, whereas some regions fail to develop effective compensatory sclerosis and instead remain in a state of persistent microdamage and insufficient repair [21]. Therefore, temporal heterogeneity in OP–OA comorbidity does not merely reflect sequential disease stages, but also reflects asynchronous entry into and exit from high-turnover states across different regions.

From a spatial perspective, remodeling imbalance is predominantly concentrated in weight-bearing regions and microdamage sites. Animal studies have shown that postmenopausal OP significantly aggravates damage to calcified cartilage and subchondral bone in weight-bearing areas, accompanied by increased subchondral osteoclast accumulation and greater porosity of the subchondral bone plate. These findings indicate that OP amplifies OA-associated bone resorption responses in locally high-stress regions [35]. This may explain why, in the comorbid state, bone resorption-dominant fragile areas, focal sclerotic regions, and bone marrow lesion areas often coexist within the same joint, rather than presenting as homogeneous remodeling. Local mechanical overload and the inflammatory microenvironment may drive uncoupled activation of osteoclasts and osteoblasts through pathways such as osteoprotegerin/receptor activator of nuclear factor-κB ligand (OPG/RANKL) and transforming growth factor-β (TGF-β), thereby further promoting site-specific remodeling responses [36,37]. Thus, subchondral bone remodeling in OP–OA comorbidity represents a focal, high-turnover, and asynchronous aberrant remodeling process established on the basis of bone fragility.

### 3.3. Coupling Between Early Bone Marrow Lesions and Cartilage Degeneration

Bone marrow lesions (BMLs) represent an important early phenotype linking aberrant subchondral bone remodeling to cartilage degeneration in OP–OA comorbidity. On magnetic resonance imaging (MRI), BMLs typically appear as ill-defined hyperintense areas on fat-suppressed T2-weighted images or fluid-sensitive sequences. Histopathologically, they correspond to bone marrow fibrosis, necrosis-like changes, microfractures, vascular proliferation, and locally increased high-turnover bone remodeling [38,39,40]. BMLs are widely regarded as important imaging markers of active subchondral bone pathology in OA. However, in the context of OP, reduced bone mass, weakened trabecular support, and decreased fatigue resistance make the subchondral bone more susceptible to microdamage and osseous decompensation under equivalent mechanical loading. Therefore, in the comorbid state, BMLs may more accurately reflect “aberrant remodeling on the basis of bone fragility” [21,41].

A clear temporal and spatial coupling also exists between BMLs and cartilage degeneration. Longitudinal studies have shown that incident, persistent, or enlarging BMLs are associated with an increased risk of subsequent cartilage loss within the same subregion, whereas reduction or resolution of BMLs is generally associated with a lower risk of local structural deterioration [42]. Moreover, BMLs may lie along a continuous pathological spectrum linking OP, OA, and subchondral insufficiency fracture (SIF). Elderly women and patients with OP are considered more prone to subchondral fragility-related injury; on MRI, such lesions often manifest as extensive abnormal bone marrow signals and may further progress to SIF, articular surface collapse, and secondary OA [21]. These findings suggest that, in OP–OA comorbidity, at least a subset of BMLs may not merely represent inflammatory or load-related signal abnormalities, but rather serve as imaging reflections of bone fragility-related decompensation, microfracture, and failed local repair.

### 3.4. Vascular and Neural Invasion and Bone Marrow Niche Remodeling

In OP–OA comorbidity, subchondral bone abnormalities are not limited to bone loss or remodeling of the subchondral bone plate; they are also accompanied by concomitant disruption of vascular, neural, and bone marrow niches. Bone vasculature and its surrounding cells jointly constitute the bone marrow vascular niche, which directly participates in osteogenesis, bone remodeling, and the maintenance of local homeostasis. This niche undergoes marked remodeling during aging and in bone-loss disorders [43]. Type H vessels are closely associated with osteogenic activity and are involved in both physiological and pathological processes in skeletal diseases such as OP and OA, suggesting that OP–OA comorbidity may share a pathological basis characterized by impaired vascular–bone coupling [44]. In OA subchondral bone, the formation of type H vessels with high expression of cluster of differentiation 31 and endomucin (CD31^hi^Emcn^hi^) is considered a bridge linking aberrant bone remodeling with cartilage degeneration [45]. Studies have shown that type H vessel formation in OA subchondral bone is closely associated with regions of active bone remodeling and is regulated by multiple signaling axes, including platelet-derived growth factor-BB/platelet-derived growth factor receptor-β (PDGF-BB/PDGFR-β), Slit guidance ligand 3/Roundabout guidance receptor 1 (SLIT3/ROBO1), transforming growth factor-β1/Smad proteins (TGF-β1/Smads), hypoxia-inducible factor-1α/vascular endothelial growth factor A (HIF-1α/VEGF-A), and mechanistic target of rapamycin complex 1 (mTORC1) [46,47,48,49,50]. Thus, vascular abnormalities are not merely secondary phenomena, but rather constitute an integral component of osteochondral unit destabilization.

An additional point that warrants emphasis is that subchondral bone alterations in OP-OA comorbidity do not always manifest as a systemic and symmetrical pattern of bone loss, but more often display marked local heterogeneity and regional specificity. Even in the setting of an overall reduction in bone mineral density, different regions within the same epiphysis may exhibit distinct patterns of blood supply, microvascular density, bone marrow lesion burden, and remodeling activity. A recent study of the human femoral head showed increased vascularization in the subchondral region of elderly patients with OA, suggesting that local vascular remodeling may participate in structural alterations of subchondral bone [51]. At the same time, studies on bone marrow lesions have shown that their genetic and histological changes are not uniformly distributed, but are closely associated with local bone remodeling, inflammatory responses, and tissue injury [52]. Therefore, local blood supply disturbance, H-type vessel remodeling, and reduced tissue microstability may together drive the coexistence of trabecular fragile regions, bone marrow lesions, and focal sclerotic areas within the same bone. The concept of tissue micro-instability proposed by Volkov et al. further suggests that osteophyte formation and local bone structural remodeling may not be determined solely by systemic bone loss, but may instead be closely related to local vascular-mechanical uncoupling [53].

On the basis of vascular abnormalities, neural ingrowth further amplifies local pathological responses. Studies have shown that, with OA progression, the expression of nerve growth factor (NGF) in osteochondral channels and bone marrow progressively increases, followed by an increase in calcitonin gene-related peptide (CGRP)-positive sensory nerves. These findings indicate that neural ingrowth exhibits clear temporal and site-specific characteristics [54]. The NGF/tropomyosin receptor kinase A (NGF/TrkA) signaling axis has been reported to drive aberrant sensory innervation and peripheral sensitization. Its activation is closely associated with the opening of osteochondral channels, increased nerve fiber density, and persistent pain transmission, suggesting that neurovascularization at the osteochondral junction is not only related to pain phenotypes but may also directly participate in the progression of structural damage [55]. In addition, osteoclast-derived Netrin-1, an axon guidance molecule, can induce abnormal extension of sensory nerve fibers into the subchondral bone, thereby establishing a molecular link among aberrant bone resorption, neural sensitization, and nerve invasion [56]. The aberrantly ingrown sensory nerve endings subsequently release neuropeptides such as CGRP and substance P (SP), which may further influence local blood flow regulation, inflammatory cell activation, and bone turnover. Therefore, neural ingrowth has dual pathological significance in both pain amplification and microenvironmental regulation [57]. Under OP–OA comorbid conditions, impairment of the barrier function of the osteochondral interface facilitates the invasion of abnormal blood vessels and nerves along porous regions of the subchondral bone plate and through osteochondral channels, thereby accelerating the transition of subchondral bone from compensatory remodeling to decompensated remodeling [58].

Bone marrow niche remodeling represents the tissue-level extension of these pathological changes. Studies have shown that OA-associated BML regions are not merely imaging abnormalities but biologically active bone marrow lesions with distinct pathological activity [59]. Single-cell sequencing has further revealed expansion of pro-inflammatory monocyte/macrophage populations, enhancement of cellular senescence phenotypes, abnormal mesenchymal cell states, and reprogramming of intercellular communication networks within these lesion areas, accompanied by upregulation of cartilage-damaging signals. These findings indicate that the bone marrow niche shifts from a relatively homeostatic reparative microenvironment to a pathological niche that continuously produces pro-degenerative signals [60]. Collectively, in OP–OA comorbidity, vascular abnormalities, neural ingrowth, and bone marrow niche disruption are not independent events; rather, they represent a continuous pathological process that drives the subchondral bone microenvironment from homeostasis toward a pro-degenerative state.

## 4. Microenvironmental Factors Driving Altered Cell Fate Plasticity

### 4.1. Abnormal Mechanical Stimulation and Altered Extracellular Matrix Stiffness

In OP–OA comorbidity, cartilage matrix degeneration and aberrant subchondral bone remodeling occur simultaneously, disrupting the mechanical continuity of the osteochondral unit. As a result, local stress transmission shifts from a relatively uniform gradient distribution to spatiotemporally heterogeneous stress concentration [61]. With the coexistence of early subchondral bone loss and subsequent focal sclerosis, the local joint environment is chronically exposed to abnormal compressive, shear, and tensile stimuli, thereby continuously reshaping the mechanical microenvironment surrounding resident cells [62]. In this context, the mechanosensitive programs of chondrocytes, osteocytes, and bone marrow mesenchymal stem cells (BMSCs) no longer maintain tissue homeostasis, but instead gradually shift toward pathological reprogramming [63]. In chondrocytes, abnormal mechanical inputs are initially sensed by mechanoreceptive structures such as cell–matrix adhesion complexes and mechanosensitive ion channels, and are subsequently converted into intracellular signaling events. Among these mechanisms, integrin-mediated cell–matrix interactions are fundamental for maintaining cartilage homeostasis. Dysregulation of this signaling axis may impair the adaptive response of chondrocytes to mechanical cues from the microenvironment, thereby promoting OA-related pathological progression [64]. In addition, the chondrocyte mechanotransduction network involves multiple regulatory layers, including Ca^2+^ channels, primary cilia, and the extracellular matrix (ECM) microenvironment. These structures collectively participate in the sensing, integration, and amplification of mechanical signals. Under abnormal loading conditions, however, mechanotransduction may shift from adaptive regulation toward pathological programs dominated by catabolism and stress responses [65]. At the downstream effector level, Yes-associated protein/transcriptional coactivator with PDZ-binding motif (YAP/TAZ) and Piezo-type mechanosensitive ion channel components 1/2 (PIEZO1/2) further mediate the conversion of mechanical stimuli into transcriptional reprogramming. Impaired nuclear translocation of YAP is associated with reduced anabolic activity in chondrocytes [66], whereas enhanced PIEZO1 mechanosensitivity can aggravate stress injury-induced cartilage degeneration [67]. Collectively, these findings indicate that abnormal mechanical stimulation persistently reshapes chondrocyte fate through multilayered mechanotransduction networks and represents an important driver of cartilage pathological progression in OP–OA comorbidity.

**Figure 1:**
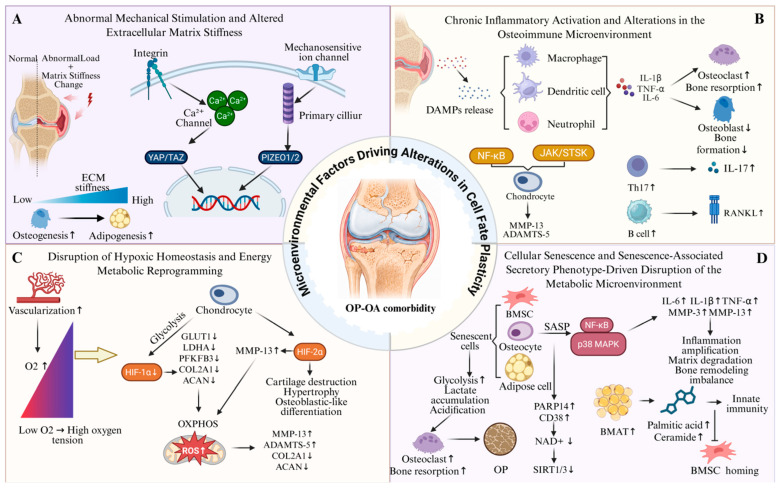
Microenvironmental factors driving alterations in cell fate plasticity. (**A**) Abnormal mechanical stimulation and altered extracellular matrix stiffness. (**B**) Chronic inflammatory activation and alterations in the osteoimmune microenvironment. (**C**) Disruption of hypoxic homeostasis and energy metabolic reprogramming. (**D**) Cellular senescence and senescence-associated secretory phenotype-driven disruption of the metabolic microenvironment.

In addition to abnormal loading, altered matrix stiffness itself acts as a critical physical signal regulating cell fate. Studies have demonstrated that matrix elasticity can directly modulate lineage commitment of BMSCs: stiffer matrices preferentially induce osteogenic differentiation, whereas softer matrices are less favorable for the maintenance of osteogenesis [68]. Subsequent studies further confirmed that matrix stiffness and cell spreading jointly regulate the balance between osteogenic and adipogenic differentiation of BMSCs. These findings suggest that regional stiffness heterogeneity within the osteochondral microenvironment in OP–OA comorbidity may promote divergent BMSC differentiation fates across different anatomical regions [69]. Therefore, in OP–OA comorbidity, low-stiffness regions caused by reduced bone mass and insufficient mineralization may weaken osteogenic signaling in BMSCs, whereas high-stiffness regions associated with focal sclerosis may amplify local mechanotransduction and cellular stress responses. Together, these processes may ultimately establish spatiotemporally divergent cell fate decisions driven by stiffness heterogeneity [70]. Overall, abnormal mechanical stimulation and altered matrix stiffness constitute important upstream mechanisms underlying osteochondral unit destabilization in OP–OA comorbidity (Figure 1A).

### 4.2. Chronic Inflammatory Activation and Alterations in the Osteoimmune Microenvironment

Chronic low-grade inflammation is one of the key mechanisms driving aberrant subchondral bone remodeling and cell fate deviation in OP–OA comorbidity. Under the combined effects of mechanical microdamage, matrix fissure propagation, and dysregulated lipid metabolism, the osteochondral interface releases damage-associated molecular patterns (DAMPs), thereby activating innate immune responses and inducing the recruitment and functional remodeling of macrophages, dendritic cells, and neutrophils [71,72]. Persistently activated immune cells release pro-inflammatory cytokines, including interleukin-1β (IL-1β), tumor necrosis factor-α (TNF-α), and interleukin-6 (IL-6). These cytokines promote osteoclast differentiation and enhance bone resorptive activity while simultaneously suppressing osteoblast function, thereby shifting bone remodeling toward a bone-resorptive state [73,74]. Meanwhile, chronic inflammation continuously activates catabolic programs in chondrocytes through key signaling pathways such as nuclear factor-κB (NF-κB) and Janus kinase/signal transducer and activator of transcription (JAK/STAT). This activation induces the production of matrix-degrading enzymes, including matrix metalloproteinase-13 (MMP-13) and a disintegrin and metalloproteinase with thrombospondin motifs 5 (ADAMTS-5), accelerating cartilage matrix degradation, tidemark disruption, and destruction of the osteochondral interface [75].

Adaptive immune responses also play a critical role in OP–OA comorbidity. Studies have shown that enrichment of T helper 17 (Th17) cells and elevated interleukin-17 (IL-17) levels in the bone marrow cavity and synovial tissue not only promote osteoclastogenesis but also enhance inflammatory crosstalk between immune cells and osteoblasts, thereby reinforcing the imbalance of bone turnover [76]. In addition, B-cell activation and B-cell-derived receptor activator of nuclear factor-κB ligand (RANKL) can further amplify osteoclastogenic signaling, weaken the microstructural stability of subchondral bone, and strengthen the capacity of the osteoimmune microenvironment to maintain a bone-resorptive phenotype [77]. As inflammation persists, immune cells also undergo metabolic reprogramming. Innate immune cells, represented by macrophages, may shift toward a glycolysis-dependent M1 pro-inflammatory phenotype, establishing a vicious cycle in which inflammatory amplification, oxidative stress, and tissue injury mutually reinforce one another [78]. Therefore, chronic inflammatory activation and alterations in the osteoimmune microenvironment are not only amplifiers of OP–OA pathological progression, but also key pathological foundations driving aberrant subchondral bone remodeling, disruption of osteochondral interface stability, and abnormal remodeling of cell fate plasticity (Figure 1B).

### 4.3. Disruption of Hypoxic Homeostasis and Energy Metabolic Reprogramming

As the barrier function of the osteochondral interface deteriorates in OP–OA comorbidity, enhanced local vascularization may alter the perfusion pattern and oxygen diffusion gradient within the osteochondral unit. This change may interfere with the hypoxia-adaptive programs on which deep-zone chondrocytes depend, thereby exposing chondrocytes to pathologically elevated oxygen tension [30]. Under physiological conditions, articular chondrocytes are chronically adapted to a hypoxic environment. Their energy metabolism is predominantly glycolytic and relies on hypoxia-inducible factor-1α (HIF-1α) to maintain the expression of glycolytic regulators, including glucose transporter 1 (GLUT1), lactate dehydrogenase A (LDHA), and 6-phosphofructo-2-kinase/fructose-2,6-bisphosphatase 3 (PFKFB3), as well as the stability of matrix synthesis programs involving type II collagen alpha 1 chain (COL2A1) and aggrecan (ACAN) [79]. By contrast, hypoxia-inducible factor-2α (HIF-2α) has been identified as a key transcriptional regulator of cartilage catabolism. It markedly promotes the expression of molecules such as MMP-13 and accelerates cartilage destruction, while also participating in chondrocyte hypertrophic differentiation and activation of endochondral ossification-like programs [80,81]. Therefore, in the comorbid state, disruption of hypoxic homeostasis weakens the HIF-1α-dominated adaptive transcriptional network while enhancing HIF-2α-mediated catabolic and terminal differentiation signals, thereby driving chondrocyte transition from homeostatic maintenance toward a degenerative phenotype.

With the loss of hypoxic homeostasis, chondrocyte energy metabolism gradually shifts from glycolytic predominance toward increased dependence on mitochondrial oxidative phosphorylation (OXPHOS), accompanied by abnormal mitochondrial membrane potential, accumulation of reactive oxygen species (ROS), and enhanced oxidative stress [82,83]. Excessive ROS not only disrupt mitochondrial homeostasis but also upregulate the expression of catabolic mediators such as MMP-13 and ADAMTS-5, suppress the expression of COL2A1 and ACAN, and ultimately weaken the matrix-maintaining capacity and stress-resistance threshold of chondrocytes [84]. Other studies have shown that HIF-1α coordinately regulates glycolysis, OXPHOS, amino acid metabolism, and lipid metabolism, making it a key node in maintaining hypoxic adaptation and metabolic flexibility in chondrocytes [85]. Meanwhile, imbalanced mitochondrial dynamics, mitochondrial DNA (mtDNA) damage, and impaired mitophagy may jointly promote chondrocyte hypertrophy, apoptosis, and extracellular matrix loss [86]. Notably, these metabolic abnormalities are not specific to OA alone. In OP, mitochondrial dysfunction is likewise characterized by impaired OXPHOS, increased ROS production, dysregulated mitophagy, and abnormal energy metabolism in BMSCs, osteoblasts, and osteoclasts. These changes can directly disturb the balance between bone formation and bone resorption while amplifying aging- and oxidative damage-related signals [87]. Thus, disruption of oxygen metabolic homeostasis may be regarded as a critical turning point through which osteochondral interface injury progresses toward cellular dysfunction in OP–OA comorbidity (Figure 1C).

### 4.4. Cellular Senescence and Senescence-Associated Secretory Phenotype-Driven Disruption of the Metabolic Microenvironment

In OP–OA comorbidity, cellular senescence is not merely an age-related phenomenon, but rather an important pathological mechanism driving homeostatic imbalance of the osteochondral unit. Studies on OA and OP have separately shown high levels of senescent cell accumulation in BMSCs, osteocytes, and bone marrow adipocytes. These changes are closely associated with impaired bone-forming capacity, expansion of bone marrow adipose tissue (BMAT), and bone loss, and may exert additive or synergistic effects within the OP–OA comorbid microenvironment [88,89]. Although senescent cells withdraw from the cell cycle, they do not enter a metabolically quiescent state. Instead, they continuously release pro-inflammatory cytokines, proteases, and growth-regulatory molecules through the senescence-associated secretory phenotype (SASP), thereby remodeling the local immunometabolic microenvironment [90]. SASP is mainly maintained and amplified through signaling pathways such as nuclear factor-κB (NF-κB) and p38 mitogen-activated protein kinase (p38 MAPK). These pathways promote the sustained secretion of interleukin-6 (IL-6), interleukin-1β (IL-1β), tumor necrosis factor-α (TNF-α), matrix metalloproteinase-3 (MMP-3), and MMP-13, thereby driving inflammatory amplification, matrix degradation, and imbalance in bone remodeling [91,92]. Single-cell sequencing studies have shown that senescent stem cell populations can aberrantly regulate the osteogenic/adipogenic differentiation balance of neighboring cells through the epiregulin/epidermal growth factor receptor (EREG–EGFR) paracrine axis. This process may predispose subchondral bone to pathological sclerosis and structural remodeling rather than effective repair [93]. Thus, OP-associated cellular senescence in epiphyseal regions may initially alter the reparative capacity and secretory profile of subchondral bone, creating persistent pathological conditions for subsequent osteochondral crosstalk and cartilage degeneration.

The pathogenic effects of SASP are further amplified by metabolic reprogramming. Mitochondrial dysfunction and nicotinamide adenine dinucleotide (NAD^+^) depletion are important foundations of senescence-associated metabolic imbalance. Upregulation of NAD^+^-consuming enzymes, such as poly(ADP-ribose) polymerase family member 14 (PARP14) and cluster of differentiation 38 (CD38), can reduce intracellular NAD^+^ levels, thereby inhibiting the activity of sirtuin 1/3 (SIRT1/3). This, in turn, weakens antioxidant defense, mitochondrial homeostasis, and matrix synthesis capacity [94]. Meanwhile, senescent cells are often accompanied by enhanced glycolysis, lactate accumulation, and local acidification. This acidic microenvironment can enhance osteoclast-mediated bone resorption and promote bone mineral dissolution, thereby amplifying bone microstructural deterioration in the context of OP [95]. Disordered lipid metabolism further reinforces the pathological effects of SASP. In OA–OP comorbidity, BMAT expansion and accumulation of lipid peroxidation products may increase lipotoxic metabolites, such as palmitic acid and ceramides. These molecules can act as metabolic damage-associated molecular patterns, activating innate immune responses, inducing metaflammation, and suppressing stem cell homing and differentiation required for osteochondral repair [96]. Accordingly, in OP–OA comorbidity, epiphyseal senescent cells and the pro-inflammatory, pro-acidifying, and lipotoxic metabolic effects shaped by their SASP are likely to serve as important mediators linking bone loss, aberrant bone remodeling, and cartilage degeneration (Figure 1D).

## 5. Cell Fate Plasticity Driven by the Subchondral Bone Microenvironment

### 5.1. Lineage Bias of Bone Marrow Mesenchymal Stem Cells and Osteogenic–Adipogenic Imbalance

During the pathological progression of OP–OA comorbidity, fate decisions of subchondral bone marrow mesenchymal stem cells (BMSCs) undergo systematic reprogramming, characterized by a shift from osteogenic toward adipogenic lineage commitment. This shift results in impaired bone formation, expansion of bone marrow adipose tissue (BMAT), and deterioration of trabecular bone architecture [97]. BMAT is a functional microenvironmental component with metabolic regulatory and paracrine activities. Its abnormal expansion is closely associated with increased bone fragility and disruption of bone homeostasis and may therefore be regarded as a histological manifestation of BMSC fate bias [98,99].

Abnormal remodeling of the metabolic microenvironment is a primary driver of BMSC lineage bias. In the comorbid state of OP and OA, accumulation of free fatty acids, enhanced lipid peroxidation, and increased oxidative stress are commonly observed. These alterations can persistently activate the adipogenic transcriptional program dominated by the peroxisome proliferator-activated receptor γ/CCAAT enhancer-binding protein α (PPARγ–C/EBPα) axis, while weakening osteogenic transcription mediated by the Runt-related transcription factor 2/Osterix (RUNX2/OSX) axis, thereby shifting BMSCs toward the adipogenic lineage [100]. Marrow adipogenic lineage precursors (MALPs), as early-stage adipogenic lineage cell populations, contribute to BMAT expansion in comorbid models. They also suppress osteoprogenitor function and amplify bone resorptive signaling through paracrine secretion of factors such as RANKL, TNF-α, and IL-6, thereby establishing a self-reinforcing “lipotoxic” microenvironmental loop [101]. In addition, aging-associated mitochondrial dysfunction and ROS accumulation preferentially impair osteogenic differentiation, which is highly dependent on oxidative phosphorylation (OXPHOS), whereas the more glycolysis-dependent adipogenic pathway is relatively preserved. This further contributes to a pathological phenotype characterized by concurrent BMAT expansion and reduced osteogenic capacity [102].

Epigenetic regulation also plays a crucial role in stabilizing BMSC lineage bias, allowing this lineage shift to evolve from a transient adaptive response into a relatively stable pathological state. The OP–OA comorbid microenvironment may induce DNA methylation and histone modification reprogramming in BMSCs. Promoter regions of osteogenesis-related genes, including RUNX2, OSX, and bone γ-carboxyglutamate protein (BGLAP), exhibit hypermethylation, whereas adipogenic genes such as peroxisome proliferator-activated receptor γ (PPARG) and fatty acid-binding protein 4 (FABP4) remain in an open chromatin conformation [103]. In particular, downregulation of histone lysine demethylases 4B and 6B (KDM4B/KDM6B) directly leads to enrichment of the repressive histone mark trimethylation of histone H3 lysine 9 (H3K9me3) at osteogenic genes, thereby epigenetically constraining osteogenic potential [104]. Long non-coding RNAs (lncRNAs) are also deeply involved in lineage determination. Loss of lncRNA Bmncr expression can relieve inhibition of the adipogenic transcription factor early B-cell factor 1 (EBF1), resulting in impaired osteogenesis of BMSCs and accumulation of marrow adiposity [105]. In addition, microRNA-188 (miR-188) and microRNA-204 (miR-204) can drive the osteogenic-to-adipogenic transition of BMSCs by targeting RUNX2 signaling [106].

**Figure 2:**
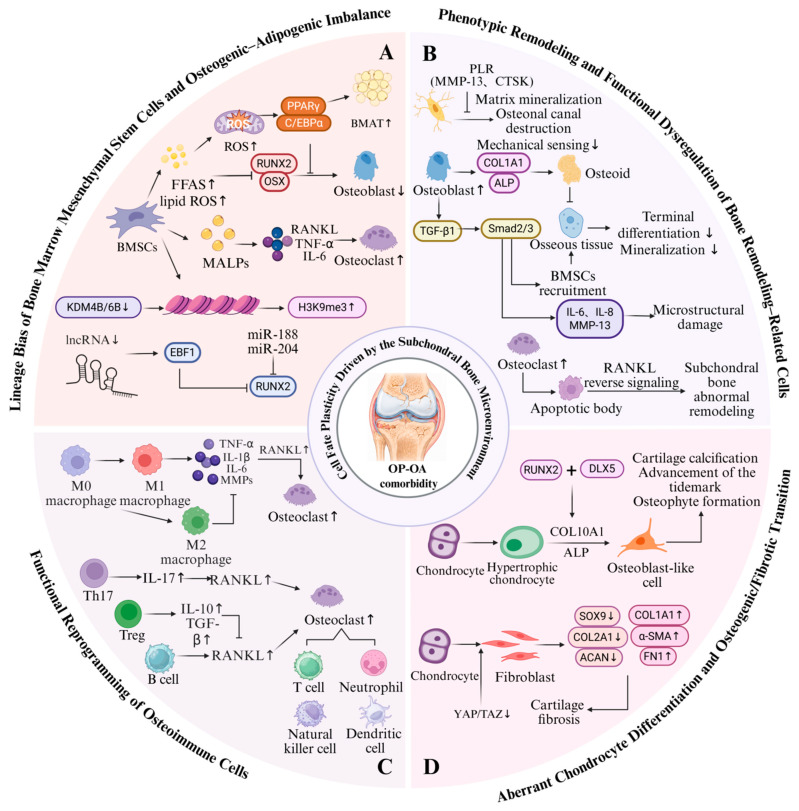
Cell fate plasticity driven by the subchondral bone microenvironment. (**A**) Lineage Bias of Bone Marrow Mesenchymal Stem Cells and Osteogenic–Adipogenic Imbalance. (**B**) Phenotypic remodeling and functional dysregulation of bone remodeling-related cells. (**C**) Functional reprogramming of osteoimmune cells. (**D**) Aberrant Chondrocyte Differentiation and Osteogenic/Fibrotic Transition.

The osteogenic/adipogenic imbalance of BMSCs not only causes bone loss but also accelerates OA progression by altering the mechanical and biochemical environment of the osteochondral interface. Expanded BMAT secretes abundant adipokines, such as leptin and resistin, which can penetrate the tidemark and directly act on chondrocytes, inducing matrix metalloproteinase (MMP) expression and cartilage matrix degradation [107]. Meanwhile, replacement of trabecular bone by adipose tissue weakens the mechanical support capacity of subchondral bone, leading to interface stress concentration and microfractures. Reduced mechanotransduction further suppresses osteogenic pathways such as YAP/TAZ and Wnt/β-catenin signaling, ultimately forming a vicious feedback loop in which OP and OA mutually reinforce one another [108]. Taken together, abnormal adipogenic bias of BMSCs may be regarded as a cellular hub linking OP and OA comorbidity (Figure 2A).

### 5.2. Phenotypic Remodeling and Functional Dysregulation of Bone Remodeling-Related Cells

The paradoxical coexistence of “bone sclerosis and bone loss” in OP–OA comorbidity can be attributed to abnormal phenotypic plasticity and functional dysregulation of bone remodeling-related cells. First, the core abnormality of osteocytes lies in impaired mechanotransduction and reduced capacity for local matrix maintenance. Osteocytes dynamically regulate the perilacunar microenvironment through the lacunar–canalicular network and matrix metalloproteinase-13 (MMP-13)- and cathepsin K (CTSK)-mediated perilacunar remodeling (PLR), thereby maintaining their mechanosensitivity. However, in OA subchondral bone, this process is markedly suppressed. Excessive mineralization of the perilacunar matrix, insufficient clearance of microdamage, and disruption of the canalicular network occur simultaneously, substantially reducing the adaptive capacity of subchondral bone under abnormal loading conditions [109]. Morphological studies of end-stage OA have revealed pronounced abnormalities in osteocyte lacunar architecture, suggesting that, in the context of OP, already fragile trabecular bone may be more prone to a vicious cycle of impaired mechanosensing and maladaptive responses [110].

Osteoblasts exhibit an abnormal phenotype characterized by enhanced osteogenic activation but impaired terminal mineralization. In sclerotic regions of OA subchondral bone, osteoblasts show active proliferation and high expression of type I collagen alpha 1 chain (COL1A1) and alkaline phosphatase (ALP). However, the matrix they secrete displays disorganized woven bone-like features, with a markedly reduced mineral apposition rate, resulting in the formation of abnormal osteoid-like tissue [23]. When abnormal mechanical loading induces excessive activation of the TGF-β1–Smad2/3 pathway, BMSCs may be aberrantly recruited and accumulated in sclerotic regions, where they contribute to osteoid deposition while their terminal differentiation into mature mineralizing osteoblasts is inhibited. This ultimately leads to the coexistence of subchondral bone plate thickening and low-quality bone [74]. In addition, OP-related systemic factors, such as estrogen deficiency, further impair the terminal mineralization capacity of osteoblasts. As a result, their response to mechanical loading shifts from a normal anabolic program toward an aberrant secretory phenotype characterized by increased production of IL-6, interleukin-8 (IL-8), and MMP-13, thereby aggravating microstructural damage in subchondral bone [111].

Aberrant osteoclast activation represents a key initiating and amplifying factor in subchondral bone remodeling in OP-OA comorbidity. In OP-OA comorbid models, abnormal subchondral bone remodeling is commonly accompanied by suppressed osteogenic and chondrogenic differentiation of BMSCs, together with enhanced migration, adhesion, and differentiation of osteoclast precursors, suggesting that osteoclastogenesis is not merely a consequence of bone loss but an active mechanism driving destabilization of the osteochondral unit. Mechanical loading intervention of the knee joint can alleviate abnormal subchondral bone remodeling by upregulating Wnt/β-catenin signaling, indicating that correction of the mechanical environment may indirectly restrain osteoclast-driven remodeling imbalance [112]. In the comorbid microenvironment, OP-related estrogen deficiency, trabecular microdamage, and disruption of the OPG/RANKL balance may prime osteoclast precursors, whereas OA-related inflammatory mediators, oxidative stress, and abnormal mechanical loading further promote RANKL/NF-κB/nuclear factor of activated T cells c1 (NFATc1)-dependent osteoclast differentiation. Recent studies have further shown that integrin beta 2 (ITGB2) participates in osteoclast differentiation and cytoskeletal remodeling in OA, thereby linking inflammatory adhesion signaling to enhanced osteoclast activity [113], whereas activation of nuclear factor erythroid 2-related factor 2 (Nrf2) can attenuate OA progression by suppressing oxidative stress and subchondral osteoclastogenesis [114]. Beyond bone resorption, osteoclasts may also function as paracrine regulatory nodes involved in cartilage, vascular, and neural remodeling. Osteoclast-derived apoptotic bodies can disturb subchondral bone remodeling and accelerate OA progression through RANKL reverse signaling [115]. In addition, osteoclast-secreted galectin-3 promotes cartilage degeneration in ovariectomized rats through the low-density lipoprotein receptor-related protein 1 (LRP1)/β-catenin axis [116], and osteoclast-derived slit guidance ligand 3 (SLIT3) may also mediate OA pain and degenerative changes, suggesting that osteoclasts do not act solely as bone-resorbing cells but may also participate in neurovascular remodeling [117]. Taken together, osteoclast-related abnormalities in OP-OA comorbidity should be understood as a combined process involving excessive bone resorption, disrupted osteoblast–osteoclast coupling, enhanced cartilage-catabolic signaling, and neurovascular remodeling. In summary, the phenotypic imbalance and mutual amplification among osteocytes, osteoblasts, and osteoclasts jointly drive low-quality subchondral bone remodeling and functional decline of the osteochondral unit in OP–OA comorbidity (Figure 2B).

### 5.3. Functional Reprogramming of Osteoimmune Cells

In OP–OA comorbidity, the subchondral bone marrow cavity becomes a pathological site where abnormal bone metabolism, chronic inflammation, and immune cell remodeling converge. OP-induced low bone mass and microdamage accumulation may enhance the interaction between subchondral bone and the bone marrow immune microenvironment, thereby continuously amplifying local immune responses associated with OA. Flow cytometric analyses have identified CD14^+^/HLA-DR^+^/CD68^+^ macrophages and CD45^+^/HLA-DR^−^/CD115^+^ osteoclast precursors in the subchondral bone marrow region of OA joints, suggesting that inflammatory cell infiltration and expansion of osteoclast precursors constitute an important cellular basis for aberrant subchondral bone remodeling [118]. In the comorbid context, estrogen deficiency, oxidative stress, failed microfracture repair, and persistent release of cartilage matrix degradation products may jointly drive osteal macrophages to shift from a homeostasis-supporting phenotype toward pro-inflammatory and pro-osteoclastogenic phenotypes [119]. M1-like macrophages release inflammatory mediators, including TNF-α, IL-1β, IL-6, and MMPs, which not only enhance chondrocyte catabolism and matrix degradation but also promote osteoclastogenesis by upregulating RANKL-related signaling. Conversely, insufficient M2-like macrophage-mediated inflammation resolution and tissue repair further establishes a vicious cycle of inflammatory amplification, enhanced bone resorption, and accelerated cartilage degeneration [120]. Therefore, in the comorbid setting, macrophage polarization imbalance should not be regarded merely as an extension of OA-associated synovial inflammation, but rather as the consequence of superimposed OP-related bone-resorptive conditions and OA-related cartilage damage signals. Single-cell sequencing studies have also shown that monocyte/macrophage populations undergo the most prominent changes after joint injury in OA, accompanied by the participation of multiple immune cell types, including T cells, B cells, neutrophils, natural killer cells, and dendritic cells. These findings suggest that subchondral bone immune remodeling is highly heterogeneous and dynamically evolving [121].

Adaptive immune abnormalities further amplify osteoimmune imbalance in OP–OA comorbidity. T cells, B cells, and dendritic cells can engage in persistent crosstalk with osteoblasts, osteoclasts, chondrocytes, and synovial cells through cytokines, chemokines, and antigen-presentation signals, thereby converting local mechanical and metabolic injury into chronic immunoinflammatory responses [122]. In OP, imbalance between T helper 17 (Th17) cells and regulatory T cells (Tregs) is considered an important immune mechanism promoting bone resorption. Th17 cell-derived IL-17 can promote RANKL expression and enhance osteoclast differentiation, whereas Tregs limit inflammation and osteoclastogenesis through interleukin-10 (IL-10) and TGF-β. When this balance is disrupted, inflammatory and mechanical injury signals in OA subchondral bone are more likely to induce osteoclast activation and disordered bone turnover [123]. B cells may also participate in osteoclast regulation through RANKL and inflammatory cytokines, thereby weakening the microstructural stability of subchondral bone [123]. In addition, in OP–OA comorbidity, osteoclasts—as effector cells derived from the monocyte–macrophage lineage—are regarded as key executors that translate osteoimmune reprogramming into structural destruction. Through bone matrix degradation, protease release, secretion of immunomodulatory factors, and neurovascular interactions, osteoclasts contribute to subchondral bone resorption, subchondral bone plate disruption, and formation of a pain-promoting microenvironment [124]. Thus, osteoimmune reprogramming in OP–OA comorbidity essentially results from the superimposition of systemic osteoclastogenic susceptibility in OP and local joint injury signals in OA, making subchondral bone a central pathological hub where immune inflammation, bone resorption, and cartilage degeneration are mutually amplified (Figure 2C).

### 5.4. Aberrant Chondrocyte Differentiation and Osteogenic/Fibrotic Transition

In the context of OP–OA comorbidity, physical and biochemical alterations in the subchondral bone microenvironment drive aberrant differentiation of articular chondrocytes, which are normally maintained in a terminally differentiated and quiescent state. First, articular chondrocytes may depart from their stable hyaline cartilage phenotype and reactivate hypertrophic differentiation and endochondral ossification-like programs. Studies based on Col10a1-Cre lineage tracing have shown that some hypertrophic chondrocytes do not inevitably undergo apoptosis, but can survive and transdifferentiate into osteoblast-like cells expressing type X collagen alpha 1 chain (COL10A1) and ALP [125]. Meanwhile, distal-less homeobox 5 (DLX5) can cooperate with RUNX2 to promote COL10A1 expression and chondrocyte hypertrophic differentiation, suggesting that hypertrophic chondrocytes may actively participate in cartilage calcification, tidemark advancement, and pathological osteophyte formation by acquiring an osteogenic-like phenotype [126]. Thus, in OP–OA comorbidity, the coexistence of systemic bone loss and local subchondral sclerosis may generate a heterogeneous mechanical state, further amplifying the positive feedback among chondrocyte hypertrophy, matrix mineralization, and aberrant subchondral bone remodeling.

Second, the transition of chondrocytes toward a fibroblast-like phenotype is another important mechanism underlying cartilage quality deterioration in the comorbid state. Under sustained mechanical overload, inflammatory stimulation, and a vascularized microenvironment, chondrocytes may gradually lose their stable hyaline cartilage phenotype and shift toward fibroblast-like or fibrocartilage-like states. Single-cell transcriptomic studies have identified a characteristically expanded fibrochondrocyte population in late-stage OA. This population shows weakened cartilage matrix-maintenance programs involving SRY-box transcription factor 9 (SOX9), COL2A1, and ACAN, together with increased expression of fibrosis-associated molecules such as COL1A1, α-smooth muscle actin (α-SMA), and fibronectin 1 (FN1). These findings suggest that chondrocytes shift from maintaining hyaline cartilage homeostasis toward low-quality fibrotic repair [127]. Other experimental evidence indicates that mechanical stress overload can induce chondrocyte dedifferentiation and acquisition of a fibrotic phenotype by inhibiting the YAP/TAZ pathway. This transition replaces viscoelastic hyaline cartilage with inferior fibrous tissue, thereby substantially weakening the anti-friction and shock-absorbing functions of the articular surface [128]. In summary, in OP–OA comorbidity, abnormal mechanical and inflammatory microenvironments drive chondrocytes toward osteogenic-like and fibroblast-like phenotypic deviations, thereby accelerating cartilage calcification, fibrotic replacement, and destabilization of the osteochondral unit (Figure 2D).

## 6. Key Signaling Networks Regulating Cell Fate Plasticity in the Subchondral Bone Microenvironment

### 6.1. Developmental Reactivation and Lineage Differentiation: TGF-β/BMP, Wnt/β-Catenin, and Hedgehog Signaling Networks

**Figure 3:**
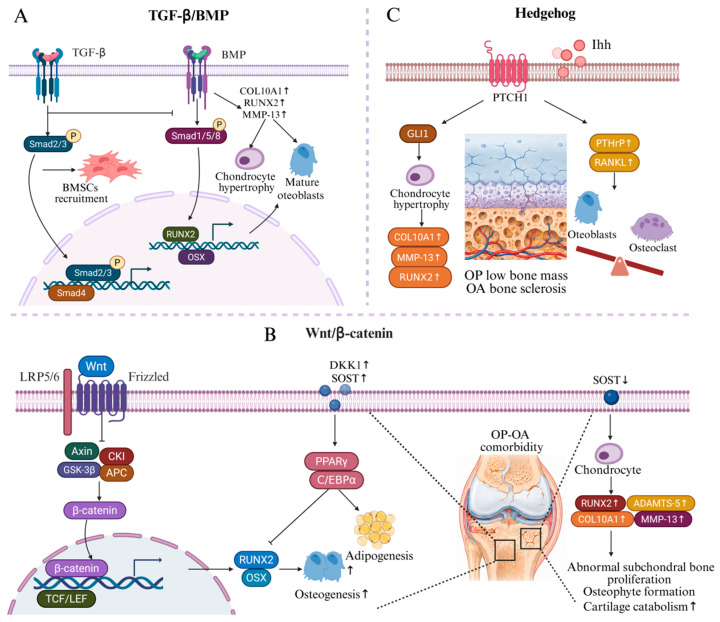
Developmental reactivation and lineage differentiation signaling networks regulating cell fate plasticity in the subchondral bone microenvironment. (**A**) TGF-β/BMP signaling network. (**B**) Wnt/β-catenin signaling network. (**C**) Hedgehog signaling network.

TGF-β/BMP signaling is primarily involved in the regulation of cartilage matrix synthesis, chondrocyte differentiation, and bone remodeling homeostasis (Figure 3A). In the subchondral bone of OP–OA comorbidity, TGF-β1 is excessively activated under conditions of abnormal mechanical loading and enhanced bone resorption. Through the Smad2/3 pathway, activated TGF-β1 recruits large numbers of BMSCs while simultaneously suppressing bone morphogenetic protein (BMP) signaling, thereby blocking the terminal differentiation of these cells into mature bone-forming cells. This process ultimately leads to the accumulation of immature osteoid-like tissue and subchondral bone sclerosis [129]. BMP signaling typically promotes osteogenic programs, including RUNX2 and Osterix (OSX), through Smad1/5/8 and is essential for BMSC osteogenic differentiation and mature bone mineralization [130]. However, when BMP signaling is aberrantly enhanced in the deep cartilage or calcified cartilage zone, it can induce the expression of COL10A1, RUNX2, and MMP-13, promoting chondrocyte hypertrophy and endochondral ossification-like transition. This dual role may partly explain the coexistence of OP-associated low bone mass and OA-associated subchondral sclerosis [131].

Wnt/β-catenin signaling is a key pathway linking the osteogenic/adipogenic fate switch of BMSCs (Figure 3B). After Wnt ligands bind to Frizzled receptors and low-density lipoprotein receptor-related protein 5/6 (LRP5/6) co-receptors, the β-catenin degradation complex composed of Axin, adenomatous polyposis coli (APC), casein kinase 1 (CK1), and glycogen synthase kinase-3β (GSK-3β) is inhibited. Stabilized β-catenin then translocates into the nucleus, where it cooperates with T-cell factor/lymphoid enhancer factor (TCF/LEF) transcription factors to activate downstream genes such as RUNX2 and OSX, thereby promoting BMSC osteogenic differentiation, osteoblast proliferation, and bone formation [132]. However, in osteoporotic regions of OP–OA comorbidity, estrogen deficiency, mechanical unloading, inflammation, and cellular senescence can upregulate Wnt antagonists such as Dickkopf-related protein 1 (DKK1) and sclerostin (SOST). These factors inhibit LRP5/6-mediated β-catenin stabilization, weakening the RUNX2/OSX-driven osteogenic program while allowing adipogenic programs mediated by PPARγ and C/EBPα to predominate. As a result, BMSCs are biased toward the adipogenic lineage [90,133]. By contrast, in locally sclerotic regions, mechanical overload may suppress SOST expression in osteocytes, thereby relieving its inhibitory effect on LRP5/6 and causing excessive activation of Wnt/β-catenin signaling. In chondrocytes, this overactivation induces the expression of RUNX2, MMP-13, ADAMTS-5, and COL10A1, thereby driving aberrant subchondral bone formation, osteophyte development, and enhanced cartilage catabolism [134,135]. Thus, Wnt/β-catenin signaling displays a typical biphasic regulatory pattern, contributing to the spatial heterogeneity of OP–OA comorbidity characterized by the coexistence of low bone mass and focal sclerosis.

The Hedgehog signaling pathway is a major driver of chondrocyte hypertrophy and endochondral ossification-like changes in OP–OA comorbidity (Figure 3C). Increased expression of pathway components, including Indian hedgehog (Ihh), Patched homolog 1 (PTCH1), and glioma-associated oncogene homolog 1 (GLI1), is associated with the upregulation of hypertrophic and catabolic markers such as COL10A1, RUNX2, and MMP-13. These findings suggest that Hedgehog signaling can reactivate developmental programs resembling endochondral ossification, thereby promoting cartilage calcification, tidemark advancement, and osteophyte formation, and further aggravating the pathological coexistence of OP-related low bone mass and OA-related focal bone sclerosis [136,137]. In addition, Hedgehog signaling can regulate the expression of parathyroid hormone-related protein (PTHrP) and RANKL in osteoblasts, thereby influencing the balance between bone formation and bone resorption. This indicates that Hedgehog signaling acts not only on chondrocytes but may also contribute to OP–OA progression by regulating high-turnover remodeling of subchondral bone [138].

### 6.2. Mechanotransduction and Cytoskeletal Remodeling: PIEZO, Integrin–FAK, and YAP/TAZ Signaling Networks

**Figure 4:**
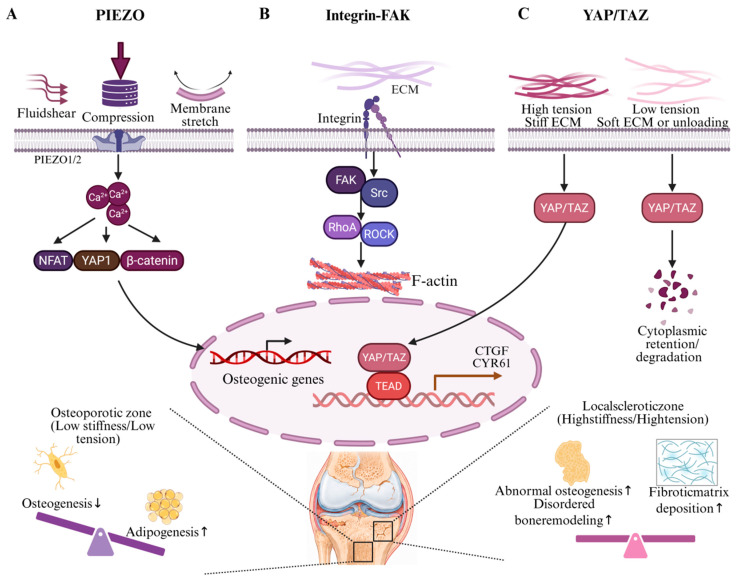
Mechanotransduction and cytoskeletal remodeling networks regulating cell fate plasticity in the subchondral bone microenvironment. (**A**) PIEZO signaling network. (**B**) Integrin–FAK signaling network. (**C**) YAP/TAZ signaling network.

In OP–OA comorbidity, mechanotransduction serves as an important bridge through which alterations in the subchondral bone microenvironment influence cell fate. PIEZO1/2 are key mechanosensitive ion channels that link mechanical stimulation to cell fate regulation. They can sense fluid shear stress, compressive loading, and changes in membrane tension, and activate downstream signals such as nuclear factor of activated T cells (NFAT), Yes-associated protein 1 (YAP1), and β-catenin through Ca^2+^ influx, thereby participating in osteogenic differentiation and bone mass maintenance. Impaired PIEZO-mediated mechanosensing leads to reduced osteogenic capacity, suggesting that PIEZO channels represent an important molecular basis for skeletal adaptation to mechanical loading [139,140]. In chondrocytes, the OA-associated inflammatory microenvironment can overactivate PIEZO1 and enhance its mechanosensitivity, making injurious mechanical loading more likely to induce Ca^2+^ overload, mitochondrial dysfunction, apoptosis, and extracellular matrix (ECM) degradation, thereby continuously driving cartilage degeneration [141]. Thus, PIEZO1/2 not only mediate adaptive osteogenic responses of bone-lineage cells to mechanical loading, but also promote aberrant subchondral bone remodeling and cartilage degeneration under abnormal mechanical conditions (Figure 4A).

Unlike the transient mechanosensing mediated by PIEZO channels, the integrin–focal adhesion kinase (Integrin–FAK) axis constitutes a key component of sustained mechanotransduction (Figure 4B). By recognizing ECM components, integrins sense matrix stiffness and adhesion status, and activate focal adhesion complex-mediated signaling pathways such as FAK/Src and Ras homolog family member A/Rho-associated coiled-coil-containing protein kinase (RhoA–ROCK). These pathways regulate actin cytoskeletal rearrangement and cellular tension, thereby stably transmitting extracellular mechanical information to the nucleus. This mechanism is essential for chondrocytes and subchondral bone cells to sense microenvironmental changes [142,143]. In osteoporotic regions of OP–OA comorbidity, insufficient matrix support and weakened adhesion signaling can reduce cytoskeletal tension in BMSCs, suppress RUNX2-related osteogenic programs, and promote their shift toward the adipogenic lineage [144]. Conversely, in locally sclerotic regions, thickened and stiffened matrix enhances Integrin–FAK signaling, inducing stress fiber formation and increased cellular contractility, thereby placing bone-lineage cells and chondrocytes in a persistent high-tension state. This sustained mechanical input not only promotes aberrant osteogenesis in subchondral bone, but may also induce chondrocyte hypertrophy, dedifferentiation, and fibroblast-like changes [145].

YAP/TAZ are key transcriptional coactivators at which PIEZO-mediated calcium signaling and Integrin–FAK-mediated cytoskeletal tension converge (Figure 4C). A stiff ECM and increased cytoskeletal tension can promote YAP/TAZ dephosphorylation and nuclear translocation, enabling them to bind transcription factors such as TEA domain transcription factors (TEADs) and activate the expression of genes related to osteogenesis, fibrosis, and matrix remodeling, including connective tissue growth factor (CTGF) and cysteine-rich angiogenic inducer 61 (CYR61). This process promotes aberrant subchondral bone formation, disordered trabecular remodeling, and fibrotic matrix deposition [93,146]. Under osteoporotic or unloading conditions, however, YAP/TAZ are more likely to remain in the cytoplasm and undergo degradation, resulting in suppression of BMSC osteogenic programs and enhancement of adipogenic differentiation [147]. In addition, the role of YAP/TAZ in chondrocytes is context-dependent. Moderate YAP activity contributes to mechanical adaptation and maintenance of matrix homeostasis, whereas in the presence of matrix stiffening and inflammatory stimulation, aberrant YAP activation may promote loss of the chondrocyte phenotype, enhanced catabolism, and degenerative progression [148]. Therefore, PIEZO, Integrin–FAK, and YAP/TAZ together constitute a mechanotransduction axis in OP–OA comorbidity, driving insufficient osteogenesis in low-bone-mass regions, aberrant osteogenesis in sclerotic regions, and cartilage degeneration.

### 6.3. Coupling Between Inflammatory Immunity and Bone Remodeling: NF-κB, OPG/RANKL, and JAK/STAT Signaling Networks

**Figure 5:**
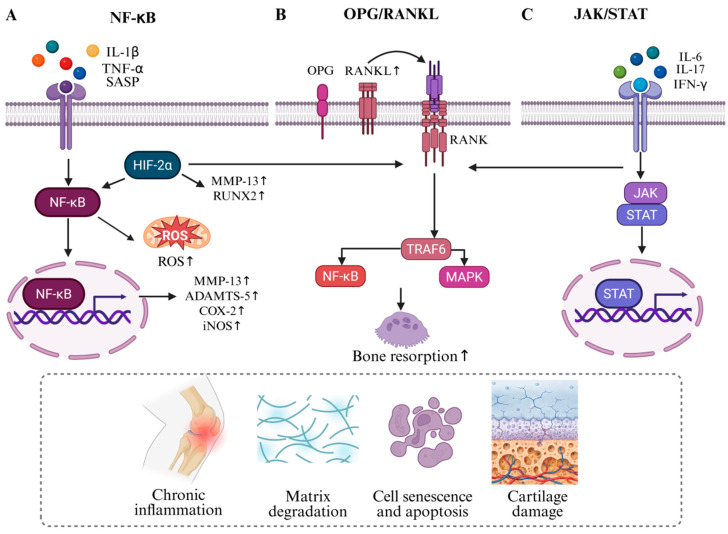
Inflammatory immune and bone remodeling coupling signaling networks regulating cell fate plasticity in the subchondral bone microenvironment. (**A**) NF-κB signaling network. (**B**) OPG/RANKL signaling network. (**C**) JAK/STAT signaling network.

In the OP–OA comorbid microenvironment, OP-associated low bone mass and disruption of trabecular microarchitecture reduce the load-bearing capacity of subchondral bone. Meanwhile, OA-related abnormal mechanical loading and matrix degradation products promote the sustained release of DAMPs, ROS, and pro-inflammatory mediators, thereby driving the osteochondral interface into a state of chronic low-grade inflammation [21]. In this environment, IL-1β, TNF-α, and SASP can activate NF-κB signaling in chondrocytes, synovial cells, and bone marrow immune cells, inducing the expression of MMP-13, ADAMTS-5, cyclooxygenase-2 (COX-2), and inducible nitric oxide synthase (iNOS). These changes promote cartilage matrix degradation and sustain inflammatory responses [149]. In addition, NF-κB can crosstalk with hypoxia-inducible factor (HIF) signaling, allowing inflammatory stimulation and a hypoxia-like metabolic state to mutually reinforce each other [150]. HIF-2α serves as a link between cartilage degeneration and subchondral bone remodeling in this process. In chondrocytes, it promotes catabolic and hypertrophic differentiation programs, including MMP-13 and RUNX2 expression, whereas in osteogenic-lineage cells, it can enhance RANKL-related osteoclastogenic signaling, thereby synergistically promoting cartilage matrix degradation and subchondral bone resorption [151]. NF-κB can also inhibit mitophagy, leading to the accumulation of damaged mitochondria and ROS bursts. This oxidative stress ultimately forces chondrocytes toward ferroptosis or senescence-associated transdifferentiation [84,152]. Therefore, in OP–OA comorbidity, NF-κB integrates OP-related subchondral microdamage, inflammatory remodeling of the bone marrow immune microenvironment, and OA-related cartilage catabolism into a continuous pathological process (Figure 5A).

The OPG/RANKL axis is the effector pathway through which inflammatory signals are converted into osteoclastic bone resorption in OP–OA comorbidity (Figure 5B). In OP, impaired function of osteoblasts, osteocytes, and BMSCs can reduce the OPG/RANKL ratio and enhance the tendency toward osteoclastogenesis [153]. Meanwhile, OA-related mechanical overload, osteocyte apoptosis, and local inflammatory cytokines can further upregulate RANKL expression. RANKL then binds to receptor activator of nuclear factor-κB (RANK) on osteoclast precursor cells, activating downstream signaling pathways such as tumor necrosis factor receptor-associated factor 6 (TRAF6), NF-κB, and MAPK. These events lead to early trabecular bone loss, porosity of the subchondral bone plate, and high-turnover remodeling in subchondral bone [154]. RANKL-mediated bone resorption not only aggravates OP-related local bone fragility, but also weakens the mechanical support provided by subchondral bone to the overlying cartilage. Furthermore, through the opening of osteochondral channels, neurovascular invasion, and formation of bone marrow lesions, it further promotes OA-related pain and cartilage degeneration [155].

The JAK/STAT pathway primarily sustains cytokine signal amplification in OP–OA comorbidity (Figure 5C). Cytokines such as IL-6, IL-17, and interferon-γ (IFN-γ) can establish paracrine loops among synovial cells, chondrocytes, and bone marrow immune cells. Through JAK/STAT signaling, these cytokines promote inflammatory mediator release, chondrocyte apoptosis, and extracellular matrix degradation [156]. Meanwhile, in OP–OA comorbidity, IL-6/JAK/STAT-mediated inflammatory signaling may further influence RANKL-related osteoclastogenic responses, transforming chronic inflammation into high-turnover remodeling and bone resorption in subchondral bone [157]. In summary, NF-κB initiates inflammatory transcription, OPG/RANKL executes osteoclastic bone resorption, and JAK/STAT maintains cytokine signal amplification. Together, these pathways drive the transformation of subchondral bone from a mechanical support structure into a pathological site characterized by persistent inflammation, osteoclastic resorption, and cartilage damage.

## 7. Limitations of Current Evidence

### 7.1. Contradictory Evidence and Interpretive Limitations

Current evidence suggests that the relationship between OP and OA is neither linear nor uniformly concordant across all patients. Epidemiological studies support a complex association between the two conditions, but this association is influenced by joint site, age, sex, skeletal region selected for bone mineral density assessment, mechanical loading status, and disease stage [158,159]. Clinically, some patients with severe osteoporosis do not exhibit overt cartilage degeneration, whereas some very elderly individuals with reduced bone mass show only relatively mild OA changes. These observations suggest that OP-OA comorbidity may be better understood as a spatially and temporally heterogeneous osteochondral phenotype, rather than a fixed pathological process determined solely by systemic bone loss.

In addition, a simple classification of certain molecular pathways as either “protective” or “pathogenic” may obscure their context-dependent roles. TGF-β, for example, contributes to cartilage homeostasis, bone formation, and tissue repair under physiological conditions, but under abnormal mechanical loading, enhanced bone resorption, or dysregulated subchondral remodeling, it may also promote aberrant bone formation, vascular invasion, and cartilage degeneration [160]. Therefore, mechanistic interpretations involving TGF-β, Wnt/β-catenin, RANKL, and inflammatory mediators should be carefully bounded by tissue compartment, disease stage, and evidence source, rather than being generalized across all OP-OA settings.

Local bone alterations and vascular factors should likewise be considered as independent dimensions of disease interpretation. In OP-OA comorbidity, skeletal abnormalities do not necessarily emerge as synchronous systemic bone loss, but may first appear as localized alterations within the epiphysis or subchondral bone. Local blood supply disturbance, abnormal H-type vessel remodeling, bone marrow lesions, and microstructural instability may generate the coexistence of fragile, sclerotic, and high-turnover remodeling regions within the same bone. Recent work on tissue micro-instability further suggests that osteophyte formation and localized epiphyseal remodeling may be associated with local vascular-mechanical uncoupling rather than being explained solely by systemic osteoporosis [53]. This concept may help explain why some patients with OP do not develop overt OA and why bone changes in OP-OA often display asymmetry and regional specificity.

### 7.2. Limitations of Experimental Models of OP-OA Comorbidity

Current experimental models remain unable to fully recapitulate the natural course of OP-OA comorbidity. In most cases, OP and OA are induced separately and then combined sequentially, making it difficult to reproduce key features such as aging, local vascular remodeling, neural invasion, bone marrow niche alteration, and intrabone heterogeneity within a single model [161,162]. For example, rabbit studies involving PTH suggest that anabolic stimulation can improve early microstructural changes, but these findings more closely reflect local modification of bone remodeling and are not sufficient to represent the long-term course of comorbidity or to explain late-stage focal sclerosis, vascular abnormalities, and pain phenotypes.

Moreover, ovariectomized rodent models mainly reflect systemic estrogen deficiency, surgical OA models primarily represent mechanically induced injury, and chemically induced models emphasize inflammatory amplification. None of these approaches fully reproduces the coexistence of bone loss, bone marrow lesions, abnormal H-type vessel remodeling, neurovascular invasion, and local subchondral bone micro-instability. Future models should therefore incorporate aging, vascular factors, and spatial heterogeneity of the osteochondral unit, and should be validated through longitudinal imaging, multi-omics approaches, and spatial tissue analysis.

## 8. Therapeutic Implications and Emerging Combined Intervention Strategies in OP-OA Comorbidity

If the core pathology of OP-OA comorbidity lies in destabilization of the osteochondral unit, therapeutic goals should move beyond simply increasing bone mass, relieving pain, or protecting cartilage alone, and instead focus on stabilizing the subchondral bone microenvironment, interrupting pathological bone-cartilage crosstalk, and improving both structural progression and symptom burden. Recent studies have further supported the concept of OA as a whole-joint disease involving cartilage, subchondral bone, synovium, bone marrow lesions, immune responses, and pain pathways, thereby providing a more rational framework for integrated intervention in OP-OA comorbidity [163]. Within this framework, subchondral bone is no longer viewed as a passive bystander, but rather as a therapeutically actionable site linking bone loss, locally increased turnover, bone marrow lesions, neurovascular invasion, and pain generation [164,165].

From a translational perspective, OP-OA comorbidity may be better managed through phenotype-based stratification rather than a uniform treatment algorithm. Patients characterized predominantly by bone marrow lesions, subchondral bone fragility, or high bone turnover may benefit more from therapies targeting bone remodeling, osteoimmune crosstalk, and neurovascular remodeling, whereas those with prominent synovitis, metabolic dysregulation, pain sensitization, or advanced structural collapse may require combinations of anti-inflammatory, metabolic, mechanical, rehabilitative, and structure-preserving approaches [166,167]. Such stratification may also help explain why single drugs or single-target interventions often show unstable efficacy in unselected populations. Future clinical studies should therefore prioritize phenotype-enriched cohorts and incorporate composite endpoints including bone marrow lesions, subchondral bone microstructure, pain, and functional outcomes in order to determine whether bone-targeted or multitarget combination therapies are truly effective in OP-OA comorbidity.

The therapeutic significance of the OP-OA concept, therefore, lies not in proposing a single universal drug, but in redefining the intervention logic. Treatment intensity should be matched to the dominant pathological phenotype, and bone protection, immune modulation, neurovascular intervention, and mechanical management should be integrated into a staged strategy. In patients with early bone fragility and high-turnover remodeling, stabilization of subchondral bone and reduction in bone marrow lesion burden may deserve priority. In those with inflammation- or pain-dominant disease, greater emphasis may be placed on modulation of the immune-neurovascular axis. In advanced structural disease, pharmacological treatment alone may be insufficient and may need to be combined with alignment correction, rehabilitation, and structural interventions.

## 9. Conclusions

OP–OA comorbidity is not a simple superimposition of bone loss and cartilage degeneration, but rather an osteochondral unit disorder centered on imbalance of the subchondral bone microenvironment. Abnormal mechanical loading, high-turnover bone remodeling, and chronic inflammation mutually reinforce one another and are accompanied by neurovascular invasion, metabolic dysregulation, and cellular senescence, gradually shifting local homeostasis toward a degenerative state. During this process, BMSCs exhibit an imbalance between osteogenic and adipogenic differentiation; bone remodeling-related cells undergo functional abnormalities; osteoimmune cells acquire pro-inflammatory and pro-osteoclastogenic tendencies; and chondrocytes undergo hypertrophic, osteogenic-like, or fibroblast-like transitions. Together, these events establish a pathological cycle of “subchondral bone microenvironment remodeling–altered cell fate plasticity–progressive osteoarticular degeneration,” suggesting that microenvironment-driven cellular plasticity is a key mechanism underlying OP–OA comorbidity progression.

However, current research still faces several limitations, including insufficient direct evidence for comorbidity-specific mechanisms, marked phenotypic heterogeneity, and unclear boundaries between cellular transdifferentiation and phenotypic remodeling. Existing animal models also remain unable to fully recapitulate the entire course of OP–OA comorbidity. Future studies should establish animal models that better reflect comorbid characteristics, combine multi-omics and spatially resolved technologies to identify specific biomarkers, and explore intervention strategies targeting the microenvironment and key cellular states. In terms of clinical translation, management of OP–OA comorbidity should shift from single-disease treatment toward individualized stratified therapy. Comprehensive treatment strategies should be developed according to bone mineral density, subchondral bone remodeling, inflammatory status, pain phenotype, and metabolic state. The combined application of antiresorptive or bone-forming therapies, osteoimmune modulation, cartilage protection, and mechanical interventions may represent an important direction for future mechanism-guided treatment.

## Data Availability

No new data were created or analyzed in this study. Data sharing is not applicable to this article.

## Abbreviations

OP	Osteoporosis
OA	Osteoarthritis
OP-OA	Osteoporosis–osteoarthritis
MRI	Magnetic resonance imaging
WOMAC	Western Ontario and McMaster Universities osteoarthritis index
PTH	Parathyroid hormone
Micro-CT	Micro-computed tomography
BMLs	Bone marrow lesions
SIF	Subchondral insufficiency fracture
CD31	Cluster of differentiation 31
Emcn	Endomucin
PDGF-BB	Platelet-derived growth factor-BB
PDGFR-β	Platelet-derived growth factor receptor-β
SLIT3	Slit guidance ligand 3
ROBO1	Roundabout guidance receptor 1
TGF-β1	Transforming growth factor-β1
Smads	Sma- and Mad-related proteins
HIF-1α	Hypoxia-inducible factor-1α
VEGF-A	Vascular endothelial growth factor A
mTORC1	Mechanistic target of rapamycin complex 1
NGF	Nerve growth factor
CGRP	Calcitonin gene-related peptide
TrkA	Tropomyosin receptor kinase A
SP	Substance P
BMSCs	Bone marrow mesenchymal stem cells
COL2	Type II collagen
ECM	Extracellular matrix
YAP/TAZ	Yes-associated protein/transcriptional coactivator with PDZ-binding motif
PIEZO1/2	Piezo-type mechanosensitive ion channel component 1/2
DAMPs	Damage-associated molecular patterns
IL-1β	Interleukin-1β
TNF-α	Tumor necrosis factor-α
IL-6	Interleukin-6
NF-κB	Nuclear factor-κB
JAK/STAT	Janus kinase/signal transducer and activator of transcription
MMP-13	Matrix metalloproteinase-13
ADAMTS-5	A disintegrin and metalloproteinase with thrombospondin motifs 5
Th17	T helper 17 cells
IL-17	Interleukin-17
GLUT1	Glucose transporter 1
LDHA	Lactate dehydrogenase A
PFKFB3	6-phosphofructo-2-kinase/fructose-2,6-bisphosphatase 3
COL2A1	Collagen type II alpha 1 chain
ACAN	Aggrecan
HIF-2α	Hypoxia-inducible factor-2α
OXPHOS	Oxidative phosphorylation
ROS	Reactive oxygen species
mtDNA	Mitochondrial DNA
BMAT	Bone marrow adipose tissue
SASP	Senescence-associated secretory phenotype
p38 MAPK	p38 mitogen-activated protein kinase
MMP-3	Matrix metalloproteinase-3
EREG-EGFR	Epiregulin/epidermal growth factor receptor
NAD^+^	Nicotinamide adenine dinucleotide
PARP14	Poly(ADP-ribose) polymerase family member 14
CD38	Cluster of differentiation 38
SIRT1/3	Sirtuin 1/3
PPARγ–C/EBPα	Peroxisome proliferator-activated receptor γ/CCAAT/enhancer-binding protein α
RUNX2/OSX	Runt-related transcription factor 2/Osterix
MALPs	Marrow adipogenic lineage precursors
BGLAP	Bone gamma-carboxyglutamate protein
PPARG	Peroxisome proliferator-activated receptor gamma
FABP4	Fatty acid-binding protein 4
KDM4B/KDM6B	Lysine demethylase 4B/lysine demethylase 6B
H3K9me3	Trimethylation of histone H3 lysine 9
lncRNA	Long non-coding RNA
EBF1	Early B-cell factor 1
miR-188	MicroRNA-188
miR-204	MicroRNA-204
MMPs	Matrix metalloproteinases
Wnt/β-catenin	Wingless/integrated-β-catenin
PLR	Perilacunar remodeling
CTSK	Cathepsin K
COL1A1	Collagen type I alpha 1 chain
ALP	Alkaline phosphatase
CD14	Cluster of differentiation 14
HLA-DR	Human leukocyte antigen-DR isotype
CD68	Cluster of differentiation 68
CD45	Cluster of differentiation 45
CD115	Cluster of differentiation 115
Treg	Regulatory T cells
IL-10	Interleukin-10
COL10A1	Collagen type X alpha 1 chain
DLX5	Distal-less homeobox 5
SOX9	SRY-box transcription factor 9
α-SMA	Alpha-smooth muscle actin
FN1	Fibronectin 1
BMP	Bone morphogenetic protein
Frizzled	Frizzled receptor
LRP5/6	Low-density lipoprotein receptor-related protein 5/6
Axin	Axis inhibition protein
APC	Adenomatous polyposis coli
CK1	Casein kinase 1
GSK-3β	Glycogen synthase kinase-3β
TCF/LEF	T-cell factor/lymphoid enhancer-binding factor
DKK1	Dickkopf-related protein 1
SOST	Sclerostin
Ihh	Indian hedgehog
PTCH1	Patched 1
GLI1	GLI family zinc finger 1
PTHrP	Parathyroid hormone-related protein
NFAT	Nuclear factor of activated T cells
YAP1	Yes-associated protein 1
Integrin-FAK	Integrin-focal adhesion kinase
FAK/Src	Focal adhesion kinase/Src kinase
RhoA-ROCK	Ras homolog family member A/Rho-associated coiled-coil containing protein kinase
TEAD	TEA domain transcription factor
CTGF	Connective tissue growth factor
CYR61	Cysteine-rich angiogenic inducer 61
COX-2	Cyclooxygenase-2
iNOS	Inducible nitric oxide synthase
OPG/RANKL	Osteoprotegerin/receptor activator of nuclear factor-κB ligand
RANK	Receptor activator of nuclear factor-κB
TRAF6	Tumor necrosis factor receptor-associated factor 6
IFN-γ	Interferon-γ
NFATc1	Nuclear factor of activated T cells 1
ITGB2	Integrin beta 2
Nrf2	Nuclear factor erythroid 2-related factor 2
Galectin-3	Galectin-3
LRP1	Low-density lipoprotein receptor-related protein 1
